# How dislocation and professional anxiety influence readiness for change during the implementation of hospital-based home care for children newly diagnosed with diabetes – an ethnographic analysis of the logic of workplace change

**DOI:** 10.1186/s12913-018-2861-z

**Published:** 2018-01-30

**Authors:** Gabriella Nilsson, Kristofer Hansson, Irén Tiberg, Inger Hallström

**Affiliations:** 1 Department of Arts and Cultural Sciences, Division of Ethnology, Lund University, Box 192, 221 00 Lund, Sweden; 2 Department of Health Sciences, Lund University, Box 157, 221 00 Lund, Sweden

**Keywords:** Professional anxiety, Time-knowledge intersection, Evidence, Paediatric diabetes, Management

## Abstract

**Background:**

In 2013–14, the evidence based care model Hospital-based Home Care for children newly diagnosed with diabetes was implemented at a large paediatric diabetes care facility in the south of Sweden. The first step of the implementation was to promote readiness for change among the professionals within the diabetes team through regular meetings. The aim was to analyse the implicit facilitators and barriers evident on a cultural micro level in discussions during the course of these meetings. What conceptions, ideals and identities might complicate, or facilitate, implementation?

**Methods:**

A case study was conducted during the implementation process. This article draw on ethnographic observations carried out at team meetings (*n* = 6) during the introductory element of implementation. From a discourse theoretical perspective, the verbal negotiations during these meetings were analysed.

**Results:**

Three aspects were significant in order to understand the dislocation during this element of implementation: an epistemological disagreement that challenged the function of information within care practice; a paradoxical understanding of the time-knowledge intersection; and expressions of professional anxiety. More concretely, the professionals exhibited an unwillingness to give up the opportunity to provide structured, age-independent information; a resistance against allowing early discharge; and a professional identity formed both by altruistic concern and occupational guardiancy. The findings suggest the necessity of increased awareness of the conceptions and ideals that constitute the basis of a certain professional practice; a deeper understanding of the cultural meaning that influences care practice within a specific logic in order to predict in what way these ideals might be challenged by the implemented evidence.

**Conclusions:**

Our main contribution is the argument that the implemented evidence in itself needs to be examined and problematized from a cultural analytical perspective before initiation in order to be able to actively counter negative connotations and resistance.

## Background

Implementing evidence-based care practice is a complex and multi-factorial process [1–3]. The importance of a better comprehension of the influences and mediators of success, and of providing a more nuanced understanding of what is going on in the reality of the implementation context, has been stressed [4–7]. PARIHS (Promoting Action on Research Implementation in Health Services) is one regularly used model for implementing evidence-based practice; a conceptual framework that considers three core elements of implementation – evidence, context and facilitation – to be dynamic and interrelated [7]. This framework suggests that successful implementation occur when evidence is robust and practitioners agree with it; the context is receptive; and where implementation processes are appropriately facilitated by internal and/or external facilitators. Rycroft-Malone and colleagues define PARIHS as a middle-range theory, meaning that it explains some of the interactions between evidence, context and facilitation whereas some, not least the role that individuals play, will remain contingent upon implementation settings, thus in constant need of improved specification in order to increase their explanatory power [8].

Though we certainly share the view on complexities and context described by Rycroft-Malone in her extensive work, from a constructivist perspective we suggest another take on contingency. Instead of aiming at minimizing or ultimately overcoming the significance of contingency, we argue that contingency must be understood as an inescapable part of all social situations and cultural processes of which care practice is one. As such, an ontological starting point, rather than a methodological problem. We challenge the objective of ever fully explaining and predicting the complexities of culturally informed human interaction. This perspective implies a methodological take that actively seeks complexity, diversity and contingency as being a prerequisite for a deeper understanding of context when implementing evidence-based care practice, rather than attempting to incorporate it into a structured model.

### Background to the study

One of the most common chronic diseases leading to lifelong treatment is type 1 diabetes. Incidence rates are increasing among children worldwide, especially in Sweden. In Sweden, initial care follows national guidelines that include one to two weeks of hospital stay for the child and one parent [9]. During the hospital stay a team of paediatricians, paediatric diabetes nurses, dieticians and social workers initiate the process of educating the parents on how to balance the child’s treatment with daily life, giving them the necessary knowledge and skills [10]. This includes longer daily meetings with the different professionals where structured information is transferred following a consistent checklist. However, parents tend to experience the theoretical knowledge acquired during the hospitalization as less valid when returning home. Among other things, they describe difficulties in handling the child’s unbalanced blood glucose levels [11, 12]. Instead parents ask for instructions that are adapted to the individual family to enable them to gradually become accustomed to their new role. Rather than teaching according to a fixed schedule, the training should be family-centered and provide time for grief and shock [13].

Based on this experience, an RCT comparing traditional hospitalization and hospital-based home care (HBHC) for children newly diagnosed with type 1 diabetes was completed 2008–2013 [14], showing that HBHC was a safe and feasible way of caring for the child, simultaneously meeting the needs of the family. Parents were reported to be more satisfied with the service in HBHC compared to hospitalization after 6, 12 and 24 months [15–17]. Additionally, some advantages to HBHC were found in the children’s metabolic control, such as fewer episodes of hypoglycaemia [18], as well as in healthcare costs reduced by 27%. Within the HBHC programme, the children received medical treatment to attain metabolic control during the first few days as inpatients if necessary. As soon as the child was medically stable, families received further care and training while staying in their home. The principal active parts of the HBHC were frequent conversations with the diabetes team to allow an individualized learning based on what was identified as the family’s current needs, as well as supporting families in their learning of management tasks in a hands-on fashion in their home environments.

## Methods

### Aim

In 2013 the process of implementing HBHC was initiated at a large paediatric diabetes care facility in Sweden. The first step in the implementation process was to promote readiness for change among the professionals within the diabetes team by repeatedly initiating reflective discussions about care practices and routines. This process was studied ethnographically. The aim was to analyse the implicit facilitators and barriers on a cultural micro level in the paediatric diabetes team meetings. What aspects of the HBHC influenced readiness for change within the paediatric diabetes team? What professional conceptions, ideals or identities might have complicated, or promoted, the implementation of this care practice?

### Design

Ethnographic fieldwork in the form of observations was conducted at team meetings (*n* = 6) held during the first element of the implementation process, before the inclusion of families. Three meetings were held in the spring and three in the autumn of 2013. The meetings lasted approximately two hours each and members from all professions participated in order to initiate reflective discussions about care practices and routines from different perspectives. Explicit problems associated with the change process, as well as with the HBHC programme, were identified and discussed. The verbal interaction between the participants, as well as other aspects evident during these meetings, was collected in a field diary which forms the empirical data.

Ethnographic research has pin-pointed the importance of observations as a method of visualizing the unspoken or even unconscious significance of common, taken-for-granted practices [19, 20]. The team meetings were selected for the ethnographic study as they are examples of concrete situations where the first element of implementation took place. The meetings are understood to be productive social situations where views and perspectives on paediatric diabetes care were produced and reproduced. More specifically, the team meetings are examples of an ongoing negotiation between the traditional, hospital-based model for care and the care practice within HBHC.

### Setting and participants

The care facility where the implementation was carried out consisted of two former separate clinics, localized to two different cities which, since 2012, had more or less willingly been merged into one unit. The work situation during the implementation process was thus marked by this major organizational change. It had been decreed that the units would work together within the same organization and apply the same care models. However, in reality the personnel still consisted of two separate teams with somewhat different routines and care practices. The observed team meetings were held variously in the two cities, with the members from both paediatric diabetes teams and the facilitator, introducing the teams to the evidence-based care practice of HBHC. The head of the facility was engaged in, and positive towards both the evidence in itself and its implementation.

The merged paediatric diabetes team consisted of a total of 24 individuals representing four professions – 10 paediatricians, 8 paediatric nurses, 4 dieticians and 2 social workers. Depending on their opportunity do leave the clinical work, to varying degrees the team members participated in the meetings. Although the intention was to get as many of the team members as possible to join the meetings, not all could participate at the same time. The number of participants at the observed meetings normally varied between 14 and 17. However, all professional groups were represented by at least one person at every meeting. Facilitating the process was the paediatric nurse who, as part of her PhD, carried out the RCT-evaluating HBHC and the traditional hospital-based care [14]. As a former employee she had gained insight and knowledge into the organizations, hierarchies and practices of the two former units. During the research and implementation process, she initially kept her inside position, but gradually changed her role from being one of the team to becoming the researcher.

### Analysis

The ethnographic data, obtained through observations during team meetings, were analysed using a discourse theoretical model [21]. This model of analysis involves a linguistic reading of what was said during these meetings. Specifically three discourse analytical concepts were applied in order to interpret the negotiations apparent during the meetings. These concepts are logic, dislocation and gripping forces, and they will be explained below.

From a discourse perspective, practices (such as care practices) are regarded as being constituted within historically, culturally and organizationally-constructed logics, logics that comprise the rules or grammar of the practice. These logics are not least manifested in professional identities and ethical ideals; in the specific context of this study, the taken-for-grantedness of what is “right”, “good” or “true” within the habitual practice of diabetes care. Applying the concept of logic in the linguistic reading of ethnographic data it becomes possible to characterize, contextualize and understand the cultural meaning and functioning of certain practices within a particular social domain, thus elucidating the transformation, stabilization and maintenance of practices. More specifically, the different care practices represented by traditional hospitalization and HBHC respectively is understood as taking place within two different logics, this simultaneously implying that practicing health care within the one logic, could make the other appear illogical.

Central in this analytical approach is the concept of dislocation [21]. Dislocation represents a breaking point that forces the subject to confront the omnipresent contingency of “truth” and “knowledge”. Dislocation is experienced as disrupting the subject’s mode of being or practising. In this study the concept was employed to interpret the implementation process as such, since HBHC demands a reorganization of both care practices and to some extent the subjectively-based professional identity of the team members. In this respect, dislocation can be regarded as something that both enforces and takes place during the implementation process, by 1) making contingency visible through the suggestion of an evidence-based alternative care practice, and 2) as it, through the presence and work of the facilitator, becomes part of an ongoing negotiation. Of particular interest from an implementation perspective is thus to examine the effects and consequences of dislocation within a certain logic.

One way to elaborate how subjects respond to dislocation on a micro-cultural level is to focus on how specific aspects tend to “grip” these subjects [21]. In order to enable comprehensive implementation, it is important to reduce the gripping forces that cause resistance and enforce the ones that increase readiness for change. Methodologically it becomes central to identify why subjects invest in certain practices and reject others; why particular practices are experienced as possible and others impossible. It will be shown through excerpts from the ethnographic data how different aspects of the HBHC model, that is the evidence in itself, grip the team members in different ways and thus effect the implementation process.

Central in ethnographic research is the hermeneutic perspective [22]. Methodologically the hermeneutic analysis implies a circular movement from the specific element in the ethnographic data to the larger contextual whole, and back again. More concrete this analytical method requires that the account for the results of the study and the independent discussion are intertwined in what has been called thick descriptions [23]. In the analytical sections below we apply this model of hermeneutic interpretation.

## Results and discussion

The results of this study suggest the necessity of increased awareness of the conceptions and ideals that constitute the basis of a certain professional practice - a deeper understanding of the cultural meaning that influences care practice within as specific logic in order to predict how these ideals might be challenged by the implemented evidence. The analysis points to the fact that certain aspects of the implemented evidence in itself might evoke connotations that become gripping forces beyond the facilitator’s reach, and thus have direct consequences for the success of the implementation.

We will highlight three examples of how micro-cultural aspects of HBHC have come to function as barriers. Firstly, the family-centred perspective on knowledge has been shown to deprive the team of the opportunity of using information as a gift that forms the basis for a contract between them as professionals and the families, within a discourse that prioritizes self-care. Secondly, the intersection between knowledge and time has been exemplified as a concrete thematic with which the dislocation was negotiated during the team meetings. This was due to the fact that a direct implication of the HBHC incentive to base the family-centred training within the home environment, was “early discharge”, something that arouses negative connotations from the history of previous organizational changes. Thirdly, expressed feelings of professional anxiety indicate that HBHC challenge the team’s professional identity. These three themes are discussed separately below, using three specific ethnographic examples from the meeting context.

The study’s strength is that it provides insight into a process of change on the cultural micro-level. Its weakness consists in that the analysis is based on one specific example alone, which makes it hard to draw generalizing conclusions. However generalization is never the objective of qualitative research, instead the study contributes to the joint scholarly body of insights about the complexity of implementation processes. As for the study as a whole, there is a risk of bias since one of the co-writers are both a researcher, the facilitator and a previous member of the diabetes team. To avoid this she was not involved in the analysis.

### Epistemological dislocation and the functioning of information as gift

An epistemological foundation of HBHC is the view of knowledge as something that must be generated gradually in relation to the family’s current understanding of the disease. Information about diabetes, and the skills to handle the disease in their daily life, should be given and practiced when it is perceived to be meaningful and constructive to the family, rather than as a consequence of the professional desire to tick off information from a checklist. This means meeting the family’s needs, but also giving information that is age appropriate in relation to the child. As seen in the ethnographic example below, this approach can be perceived as problematic:

The team discusses how to relate to information that is categorized as “very important” but perhaps not age appropriate. The effects of drinking alcohol and smoking are mentioned as examples. The team expresses the view that even though knowledge about the risks associated with drinking and smoking might not be relevant to the parents of an eight year old at present, this knowledge is still considered to be something important for them to “take home” from the hospital. It is always good “to have mentioned” certain things. One member of the team stresses the need of some kind of tool to use to be able to keep track of what has been mentioned and what has not. She suggests the use of a form to make sure that all team members are aware of where, in the process of learning about diabetes, the family is. Could it be entered into the medical journal? The facilitator rejects this by explaining that the new perspective is all about objectives, not about specific subjects of information. The team should guide the families, not lecture them. Their work should be about trying to acknowledge where the families are, and where they are heading (Team meeting, 8 April 2013).

This excerpt represents one example of how the care practice, implied by the implementation of HBHC, appeared disturbing to members of the professional team. The requirement that the team actively omit “very important” information leaves them concerned about what might happen to the children in the future. It is evident that specifically the risks of drinking alcohol and smoking become gripping forces that motivate some team members to argue for, and invest in, the traditional care practice stating that some things are just good “to have mentioned”. One way to analyse these concerns is as a consequence of the team members’ conceptions on risk. Diabetes is in fact a disease that imposes serious requirements as concerns medical knowledge in order for the individual to live in a way that counteracts the risk of complications. One obvious task for the health care professions is to counteract medical problems. However the societal call on the individual to act “rationally” upon encountering risk, and as a consequence the professionals’ efforts to educate the citizens to become responsible individuals, is a discourse that has influenced the understanding of health in the western world in a profound manner since the early 1900s [24, 25]. The team members’ concerns must be understood in relation to this discourse. Leaving out certain information might be perceived as actively putting the child at risk, contradictory to the team’s professional ethics. Based on this we would argue that implementing HBHC not only implies changes in care practices, but something that also affects their professional identity.

Another way to describe the concerns expressed in the meeting context exemplified above has to do with epistemology, more specifically the team’s view on what knowledge is and how it is achieved. HBHC implies a major change of conception, from a linear, information-based view on knowledge, to a process-based relational view. Though this perspective is in line with contemporary pedagogical conceptions, the decree that their care practice should be “all about objectives, not about specific subjects of information” becomes a dislocation in the sense that it enforces the team members to acknowledge contingency in paediatric diabetes care. Instead of checking off information points from a form, they need to be responsive and flexible to the families’ immediate needs. The contradictory proposal by one of the members to actually create a new form to fill in regarding the information the family has been given can be regarded as an attempt to negotiate with this dislocation. This reaction must be explained within its specific logic, in this case what we would describe as a logic of professional responsibility and health care ethics.

Historically, work in health care and medicine has been guided by ethical guidelines and ideals where “protecting the patient’s best interest” is of central importance. In Sweden, these ideals are explicitly articulated in the Swedish Health and Medical services Act and can be assumed to be deeply rooted in the identity of healthcare professionals, constituting a joint feeling of professional responsibility. In recent decades, the basis for this care logic has been increasingly challenged by the self-care discourse in the form of policy changes that further relocate responsibility for the patient’s health and wellbeing, from the professional to the patient, as well as introducing new principles for cost efficiency [26]. The key component in HBHC – that the families should gradually be given the tools to make informed decisions in their everyday life, rather than be kept dependent on professional medical supervision – is in line with this logic of self-care. Investing in defending the traditional epistemology could, on one level, be regarded as resistance to this discourse. However an elaborated analysis on why particularly information is given such a central position in the team’s view on good paediatric diabetes care might enable a partially different interpretation. In pursuing this analysis we will reflect on the cultural function “checking off information” fulfils in the team members’ interaction with the families.

Evident in the example above, information seems to be viewed as something possible to “give” and, when given, something that the families “have” – namely something they “take home” with them when they leave the hospital. In cultural theory the act of giving and the gift itself has been of recurrent interest as a way of understanding social interaction. In his classical work, cultural anthropologist Marcel Mauss [27] describes the symbolic meaning and cultural function of a gift to be twofold. A gift creates a shared interest in a specific matter, but simultaneously places this reciprocity in a power relationship in the sense that a gift entails a duty to receive the gift, as well as a duty to repay it. From this perspective, by giving the families information on how they should counteract complications, the team members indirectly require them to both assimilate the information into active knowledge (receive it) and to reciprocate the gift by living in accordance with its recommendations. We argue that information could be regarded as the basis of a contract between the health care professionals and the families. Without the function of information as a gift (as implied when implementing HBHC), the team loses the opportunity to establish the contract with the families, the contract that imposes demands on their level of self-care. This suggests that resistance against HBHC should not necessarily be regarded as criticism of the self-care logic per se, but rather against being deprived of one of the most important tools, or strategies, with which the traditional care logic can be fitted into this self-care logic. As stated by Castellani and Hafferty [28], agents (in our case the team members) in a complex social system use the web of subsystems to create, organize and change the system in response to the demands of the external environment. This analysis points to the fact that certain aspects of the evidence implemented in itself might evoke connotations that become gripping forces beyond the facilitator’s reach, and thus have direct consequences for the success of the implementation. In the next section a concept culturally similar to information and knowledge will be discussed, namely time. It will be argued that the approach to time in HBHC might trigger previous, negative experiences of change among the team members, and thus function as a barrier.

### Time-knowledge intersection

As described above, HBHC implies individualized (family-based) care, where continuous learning is central. It has been argued that this approach constitutes an epistemological dislocation for the team members in that they will no longer give information in a structured manner, but offer relevant information when requested by the families themselves. However, this dislocation does not solely derive from the epistemological shift, but rather from the intersection between knowledge and time. This is due to the fact that HBHC simultaneously opens up the possibility of shortened hospital stays. The two active components, a more protracted learning process combined with a shortened hospital stay, are perceived as paradoxical by the members of the team. In the following longer quote, we wish to give one example of how this paradox is negotiated:

Diabetes Nurse 1: My experience is that it takes a couple of talks before we understand what level the family is on. It helps a bit to ask about their level of education, but with the shock and everything, it’s difficult to assess where they are, in the beginning. Usually it is when they start asking questions that you get an understanding of that. That they haven’t even understood if the insulin raises or lowers the blood sugar.

Social worker 1: And that might take three or four days.

Paediatrician 2: I mean, we want to do it well and it’s stressful to feel that we need to check off so many things. But we don’t have to think like that anymore. As I did before, I gave them so much information, but rather as you say try to understand it from the families’ perspective. To sit down and take it really slowly and see what comes up.

Diabetes Nurse 2: But at the same time it is difficult to combine this approach with the fact that they shouldn’t stay at the hospital longer than necessary. In the past it was possible to do it this way. The families were there and we could wait until they were ready before we started to give them any information. But now it’s like discharge from hospital as soon as possible. And by then they just have to know certain things; how to give insulin and how to handle hypoglycaemia. So you just have to go over that, whether they are ready or not. And that’s what is so hard.

Paediatrician 1: Yes, I agree with you on that. Discharging them as soon as possible can’t be what’s most important here. We have families that don’t want to stay more than 24 h, like my last two cases, but others need to stay a week or more because they are just too jittery. It should be individual I think, how long they stay. It differs so much between families. One can be very calm about it from the start, and for another it’s really chaotic. And it might still be chaotic after a week.

Facilitator: Yes, but my experience [from the RCT] is that with this new approach, it takes the parents about two days and after that they were pretty independent. Actually I think I was the major impediment, which made me think a lot. You talk about the state of shock they are in, but isn’t it a fact that they are consumed by certain thoughts at this stage? If we try to talk about something else, they’re not present, but if we can capture what’s on their minds, I believe it is easier to get the family to focus.

Social worker 2: Yes, I agree with you. But the problem is that we all have our agendas, so to speak, with the things they need to know before they are released. I mean, they really need to know certain things before they leave the hospital.

This excerpt offers one example of how opportunities and problems are under constant negotiation during the team meetings observed. One central feature in this case is that the dislocation seems very concrete. The team members cannot comprehend how the family-centred learning process could be combined with a shortened hospital stay. To “sit down and take it really slow and see what comes up”, as described positively by one of the paediatricians, is perceived by some of the others to be “difficult to combine […] with the fact that they [the families] shouldn’t stay at the hospital longer than necessary”. The amount of information believed to be possible to impart to the families seems closely related to the number of days they are admitted to the hospital, in the view of the team members. It is apparent that their concerns about (lack of) knowledge are closely connected to their experience of (lack of) time. How is it possible to meet the needs of the patients, if there is no time to wait for them?

Central to HBHC is the hands-on perspective on how knowledge is optimally accumulated, namely within an everyday context in the home environment. In practice this means that the families can be discharged from the hospital before they have received all the available information about caring for a child diagnosed with diabetes. Waiting them in, so to speak, does not require that the family remain at the hospital. This, however, articulates discrepancies in the view on the knowledge that is absolutely necessary before the families are released from the hospital; more specifically before the parents are trusted with the responsibility for the child’s health. This is evident in the quote from one of the social workers: “I mean, they really need to know certain things before they leave the hospital”. The team, in a somewhat frustrated manner, argues for the impossibility of ever overcoming this paradox in two ways. Firstly, they use descriptions of the families’ actual “being” i.e. in shock, chaotic, jittery as evidence that it takes time before they can even begin teaching them about diabetes care. In this state of being they might not even understand “whether the insulin increases or lowers the blood sugar”. Secondly, their different professions are described as competitors for this time – “we all have our agendas, so to speak”. These emotionally-charged arguments reveal that they are investing in the defence of the current care practice.

This example, however, does indicate that frustration is not necessarily derived from the implementation of HBHC specifically. Instead the discussion implicitly refers to a joint experience of previous (organizational) changes of the conditions for practicing diabetes care. This is evident in the quote by one of the nurses where an explicit distinction is made between the past and the present: “In the past it was possible to do it this way [as suggested in HBHC]. The families were there and we could wait until they were ready before we started to give them any information. But now it’s like: discharge from hospital as soon as possible”. It is not clearly expressed here who would be responsible for this change, or when it was carried out. Instead, it breathes a general criticism directed at a political system that prioritizes financial incentives for self-care; a system that is thought to force the team to impart information to the families before they are ready to receive it. Hence, to understand the team’s reaction towards the dislocation of time imposed by HBHC, this reaction must be viewed from within a logic formed by a history of demands for shortened hospital stays. That is, once again, as a response to a societal discourse of self-care as well as a new managerial perspective.

Many western countries are struggling with increased costs for health care services. With regard to hospital organization, this context has been described as reinforcing a bureaucratic rationalism, with scientific management being given more power to ensure that hospital costs are better controlled and more predictable. Scholars from medical humanities have indicated the contemporary emergence of more complex professional roles that demand control of both managerial tools, involving funding and efficiency, and organizational tools that guarantee quality and safety [29]. From within a logic accustomed to this history of policy change, HBHC can be perceived as part of this change. Negative connotations towards “early discharge” apparently function as a gripping force that impacts the team’s readiness for change. These connotations might thus function as a barrier to implementing HBHC. This both in the sense of the nurse above, namely that the suggested care practice would not in fact be possible anymore, and in form of general resistance against further changes in this direction.

However, drawing from the same excerpt we suggest that their criticism of the “early discharge” trend could have worked in the opposite direction thus, on the contrary, facilitating readiness for change. This is indicated by how some of the team members use central components of HBHC as supportive arguments against early discharge, namely the aspect of family-centred care. This is most evident in the quote by one of the paediatricians, stating that “discharging them as soon as possible can’t be what’s most important here. […] It should be individual I think, how long they stay. It differs so much between families”. We argue that this quote should be interpreted as an invitation to negotiate the intersection between time and knowledge within HBHC – a reaching out directed at the facilitator participating in the discussion. A response to this invitation, by acknowledging the team’s frustration over certain aspects of the care policy history, might have triggered positive feelings towards HBHC that in turn could have facilitated its implementation. The facilitator in this situation, however, does the opposite declaring that there should be no modification of the relationship between time and knowledge; this by stating that “my experience is that with this new approach, it took the parents about two days and after that they were pretty independent”. Here evidence explicitly outdoes professional feelings and experience. In the next section this issue of emotions is further elaborated with the concept professional anxiety.

### Professional anxiety

In the previous sections we emphasized the dislocation caused by the implemented evidence, that is, the demands for a new perspective on “good” paediatric diabetes care. In this final section we wish to elaborate the emotional expressions related to this dislocation; expressions such as frustration, worry and even fear. To a great extent these feelings have to do with concerns for the child’s wellbeing. Central within health care is obviously to care for the person seeking help. However, we would like to add to this assumption by suggesting yet another potential ground for these emotions; this has to do with professional legitimacy. In order to analyse both these potential reasons for emotional expression simultaneously, we will apply the concept “professional anxiety” to which we ascribe two intertwined meanings – altruistic concern and occupational guardiance.

First it becomes necessary to reflect on how professionalism functions in the life professions. Occupational theory has described a changing view on what constitutes a profession and what drives professionalization processes; from an expert ideology driven by progress optimism and altruism in the 1940s onwards, to a view in the 1970s that focused on professionalization as a process of occupational guardiance, dominated by knowledge monopolies and closure regimes [30]. Since then, both perspectives have been challenged. Instead the context-sensitivity of any profession and professionalization process has been stressed [31]. It is noted that the relationship between the professional and the patient has changed to the extent that the professional’s knowledge monopoly is no longer obvious – the classical “expert” has become rarer within the self-care discourse. The home-based starting point for HBHC is in line with this change. Nonetheless it is likely that both the perspectives of altruism and guardiance are active components in what constitutes a certain professional identity [32].

In order to explain the cultural meaning of professional anxiety it becomes necessary to depict the responsibilities that constitute the basis for the team members’ professional identities. One central ideal in medical professionalism is not only the knowledge and adherence to principals and duties but also the development of a virtuous and moral character in which altruistic concern are habitual and defaulted to even in times of significant stress [33]. Feelings of professional anxiety are thus closely interconnected with uncertainties regarding who, in the end, is responsible for the child’s health. This is exemplified through the fact that the team’s concerns are particularly strong in relation to families who, for different reasons, are thought of as less able to act in a responsible manner. Families are described as reacting differently to the diagnosis – some relate to it constructively and are, from the start, susceptible to the information given, whereas others are described as falling into a state of shock where they cannot assimilate the information without panicking. It is plausible that the dislocation caused by implementation reinforces feelings of a gap between professional liability and the perception of the parent’s accountability. The use of information as a gift, as described above, can be regarded as a strategy to counteract this gap. However, in order to fully understand the basis of this professional anxiety we must take into account the fact that the team’s opinions on the parents not only consist of assumptions on where they are in the process of learning how to manage the child’s diabetes, but also who they are in terms of social categorization [34]. As formulated by one of the paediatricians: “I think we need to consider what type of family we have to deal with”. Some families are understood as well suited for the new care model, whereas others are considered to be more problematic - some just do not seem to follow recommendations.

Among risk sociologists, risk is thought of as closely connected to trust; a person’s experience of safety is when there is a balance between trust and risk. Differently put, showing trust can be thought of as putting risk within brackets, as if it did not exist [35]. In hospitalization the patient experiences safety by putting trust in the professionals’ ability to counteract risk. However with HBHC, the opposite situation is required: the team members are forced to put risk within brackets and trust the families’ ability. This, on the one side, challenges their altruistic concern and might result in feelings of frustration and resignation [36]. This is apparent in a quote from one of the paediatricians, "For some it feels pointless. They need support to make it work in their everyday life. Those with a high level of HbA1c, we haven’t reached them. If we had, they wouldn’t have had that level".

But on the other side, and equally important, is the fact that HBHC flips the roles in the relationship between care giver and care taker, private and professional. To some extent the parents become the experts on their everyday life and the team members the novices. This is visible in the excerpt presented in the previous section where the facilitator stated that she was the biggest problem when it came to releasing the families: “Actually I think I was the major impediment, which made me think a lot”. From a perspective on professionalization as dominated by occupational guardiance, knowledge monopolies and closure regimes, feelings of professional anxiety can be seen as a consequence of the need to defend the professional identity. Strategies of resistance, as discussed above in relation to the gift, could be analysed as a way to recreate a sense of professionalism, and thus counter professional anxiety on the individual level.

Another aspect of professionalism is that its legitimacy is co-constructed with the amount of trust that is placed in the system in which the professional and the private individual interact, in this case the very organization that provides care. This trust is, in turn, based on the expectations of different roles and practices, i.e. that the professionals act in the way they are expected to do – they provide care. It is plausible that HBHC is thought to impair the opportunities to meet the expectations upon which their professional’s legitimacy rests. This is not least true in a time were professionalism is not to the same extent legitimized by norms on the organizational and societal level, but something that is constructed and articulated on the individual level in the concrete care practice [37].The relationship between professional and patient is still characterized by a mutual recognition of the professional’s skills, but the conditions for how professionalism can be exercised have changed.

## Conclusion

The purpose of this qualitative ethnographic study was to extend the medical/health care implementation debate by providing an insight into wider cultural argumentation. Our main contribution is the argument that the implemented evidence in itself needs to be examined and problematized from a cultural analytical perspective before onset in order to be able to actively counter negative connotations and resistance. Building a more effective model for implementation requires careful evaluation of the cultural characteristics of the implemented evidence. In other words, trying to predict the dislocation and counteract the gripping forces triggered by this dislocation. This means actively asking cultural analytical questions before initiation. What norms and conceptions does this evidence challenge? What memories of previous experiences might it trigger? What individual threats does it raise? To do so, we need to learn the “history of practice” challenged by evidence, on the local level as well as on the societal level. In this study we have argued that professional anxiety must be understood in the context of societal change, specifically formed by the emergence of a self-care discourse. This includes pinpointing the “right”, the “good” and the “true” within the logics of the professional groups affected.

## Abbreviations

HBHC: Hospital Based Home Care
PARIHS: Promoting Action on Research Implementation in Health Services
RCT: Randomized Controlled Trial
